# Supplementary material for: Cost-benefit analysis of a multicomponent breastfeeding promotion and support intervention in a developing country
Source: PLoS One. 2024 Jul 19;19(7):e0295194. doi: 10.1371/journal.pone.0295194 (PMC11259277; doi:10.1371/journal.pone.0295194)
Supplement: S1 File — (PDF) [file pone.0295194.s001.pdf]

S1 File. Comparison of the trial's experimental and the control groups at different time points.

	**Control**	**Experimental**	**Unadjusted Analysis**	**Adjusted Analysis[1]**
**CATEGORICAL VARIABLES**	***n (%)***	***n (%)***	****p-Value***	***p-Value***
**Doctor visit for infant illness**				
**Month 1**	42 (23.5)	28 (17.5)	0.176	0.168
**Months 2 and 3**	53 (31)	36 (22.9)	0.101	0.250
**Months 4 to 6**	74 (46)	63 (42.6)	0.548	0.497
**First 6 months**	110 (68.3)	92 (62.2)	0.256	0.291
**Months 7 to 12**	103 (65.2)	89 (62.7)	0.595	0.835
**First 12 months**	133 (84.2)	122 (85.3)	0.784	0.624
**Months 13 to 24[2]**	118 (77.1)	112 (83)	0.218	0.961
**First 24 months[2]**	141 (92.2)	132 (97)	0.071	0.254
**Infant hospitalization**				
**Month 1**	10 (5.6)	10 (6.3)	0.796	0.855
**Months 2 and 3**	7 (4.1)	7 (4.5)	0.870	0.879
**Months 4 to 6**	9 (5.6)	4 (2.7)	0.207	0.243
**First 6 months**	19 (11.8)	17 (11.5)	0.931	0.771
**Months 7 to 12**	20 (12.7)	8 (5.6)	**0.035**	0.085
**First 12 months**	33 (20.9)	22 (15.4)	0.217	0.256
**Months 13 to 24**	15 (9.8)	18 (13.2)	0.360	0.307
**First 24 months**	43 (28.1)	34 (25)	0.551	0.647
**CONTINUOUS VARIABLES**	***Mean (SD)***	***Mean (SD)***	***[£]p-Value***	***p-Value***
**Number of doctor visits for infant illnesses**				
**First month**	0.25 (0.495)	0.26 (0.666)	0.939	0.998I
**Months 2 and 3**	0.45 (1.091)	0.34 (0.739)	0.278	0.664

	Control	Experimental	Unadjusted Analysis	Adjusted Analysis[1]
**Months 4 to 6**	0.83 (1.551)	0.54 (0.999)	0.054	0.084
**First 6 months**	1.50 (1.982)	1.13 (1.527)	0.062	0.134
**Months 7 to 12**	1.49 (2.312)	1.13 (1.448)	0.096	0.154
**First 12 months**	2.97 (3.095)	2.22 (2.302)	**0.016**	**0.045**
**Months 13 to 24[2]**	2.77 (3.876)	2.83 (3.990)	0.900	0.994
**First 24 months[2]**	5.75 (5.718)	5.04 (5.069)	0.275	0.322
**Number of infant hospitalizations**				
**First month**	0.06 (0.230)	0.06 (0.243)	0.797	0.858
**Months 2 and 3**	0.04 (0.199)	0.06 (0.283)	0.542	0.446
**Months 4 to 6**	0.06 (0.230)	0.03 (0.163)	0.202	0.238
**First 6 months**	0.12 (0.349)	0.14 (0.398)	0.789	0.887
**Months 7 to 12**	0.15 (0.420)	0.08 (0.337)	0.117	0.192
**First 12 months**	0.27 (0.583)	0.21 (0.567)	0.349	0.401
**Months 13 to 24**	0.17 (0.667)	0.16 (0.443)	0.904	0.999
**First 24 months**	0.45 (1.026)	0.37 (0.758)	0.438	0.527
**Total cost of formula and water (USD)**				
**First month**	22.91 (33.929)	22.50 (36.593)	0.915	0.373
**Months 2 and 3**	87.94 (105.561)	94.54 (110.771)	0.581	0.782
**Months 4 to 6**	173.51 (167.921)	171.09 (174.993)	0.902	0.411
**First 6 months**	284.51 (282.091)	283.57 (286.489)	0.977	0.422
**Months 7 to 12**	303.92 (278.559)	367.46 (306.804)	0.061	0.473
**First 12 months**	581.08 (486.212)	641.56 (528.693)	0.302	0.938
**Months 13 to 24[2,3]**	559.98 (396.350)	583.43 (395.096)	0.617	0.942
**First 24 months[2,3]**	1156.11 (722.304)	1224.45 (729.816)	0.427	0.826
**Total cost of doctor visits for infant illnesses (USD)**				
**First month**	9.02 (21.402)	10.38 (33.172)	0.649	0.955
**Months 2 and 3**	19.69 (54.461)	13.98 (35.543)	0.266	0.266

	Control	Experimental	Unadjusted Analysis	Adjusted Analysis[1]
**Months 4 to 6**	33.45 (63.808)	26.44 (56.773)	0.311	0.299
**First 6 months**	61.52 (85.898)	51.35 (82.856)	0.291	0.194
**Months 7 to 12**	64.01 (103.932)	55.37 (78.547)	0.420	0.328
**First 12 months**	125.02 (141.429)	102.92 (120.910)	0.148	0.101
**Months 13 to 24[2]**	133.97 (221.802)	144.40 (217.761)	0.688	0.840
**First 24 months[2]**	259.59 (308.066)	249.44 (265.564)	0.766	0.398
**Total cost of infant hospitalizations (USD)**				
**First month**	793.82 (4,293.041)	886.00 (4,492.518)	0.847	0.735
**Months 2 and 3**	604.22 (3,049.150)	948.43 (5,050.373)	0.451	0.447
**Months 4 to 6**	628.34 (2,603.148)	515.62 (3,775.232)	0.759	0.876
**First 6 months**	1,526.38 (4,535.873)	1,771.35 (5,643.984)	0.673	0.730
**Months 7 to 12**	1,835.65 (6,567.496)	982.74 (4,709.101)	0.193	0.319
**First 12 months**	3,391.02 (8,298.904)	2,714.96 (8,077.900)	0.475	0.577
**Months 13 to 24**	2,876.90 (1,3049.830)	2,780.44 (9,805.611)	0.944	0.998
**First 24 months**	6,378.74 (18,766.041)	5,481.24 (12,630.570)	0.638	0.726
**Total cost of infant medications (USD)**				
**First month**	2.61 (12.960)	4.19 (21.314)	0.404	0.448
**Months 2 and 3**	6.07 (19.648)	4.01 (13.848)	0.275	0.318
**Months 4 to 6**	12.92 (40.289)	6.77 (15.313)	0.073	0.088
**First 6 months**	20.91 (55.627)	13.64 (22.971)	0.140	0.159
**Months 7 to 12**	28.05 (64.557)	18.67 (52.137)	0.169	0.251
**First 12 months**	43.75 (71.534)	31.16 (59.276)	0.099	0.161
**Months 13 to 24[2]**	28.39 (39.335)	31.20 (59.433)	0.633	0.583
**First 24 months[2]**	72.15 (92.296)	62.04 (94.675)	0.360	0.444
**Total cost of maternal non-routine doctor visits due to BF (USD)**				
**First month**	3.49 (17.443)	3.88 (21.634)	0.853	0.513
**Months 2 and 3**	2.79 (12.502)	2.18 (13.200)	0.665	0.584

	Control	Experimental	Unadjusted Analysis	Adjusted Analysis[1]
**Months 4 to 6**	2.75 (17.748)	1.25 (9.268)	0.358	0.208
**First 6 months**	8.98 (36.528)	7.76 (28.226)	0.745	0.300
**Months 7 to 12**	1.47 (16.060)	0.09 (1.111)	0.283	0.332
**First 12 months**	10.62 (45.280)	8.12 (28.676)	0.573	0.241
**Months 13 to 24**	8.87 (49.056)	2.18 (15.552)	0.112	0.091
**First 24 months**	19.84 (84.309)	10.72 (32.644)	0.218	0.094
**Overall total cost[4] (USD)**				
**First month**	831.84 (4308.943)	970.56 (4496.961)	0.772	0.793
**First 6 months**	1902.30 (4601.112)	2389.28 (5706.914)	0.408	0.510
**First 12 months**	4151.48 (8372.374)	3760.34 (8180.059)	0.683	0.725
**First 24 months[3,5]**	7862.77 (18922.603)	7373.06 (12843.583)	0.801	0.838
	**Control**	**Experimental**	**Unadjusted Analysis**	**Adjusted Analysis[1]**
**CATEGORICAL VARIABLES**	***n (%)***	***n (%)***	***p-Value***	***p-Value***
**Doctor visit for infant illness**				
**Month 1**	42 (23.5)	28 (17.5)	0.176	0.168
**Months 2 and 3**	53 (31)	36 (22.9)	0.101	0.250
**Months 4 to 6**	74 (46)	63 (42.6)	0.548	0.497
**First 6 months**	110 (68.3)	92 (62.2)	0.256	0.291
**Months 7 to 12**	103 (65.2)	89 (62.7)	0.595	0.835
**First 12 months**	133 (84.2)	122 (85.3)	0.784	0.624
**Months 13 to 24[2]**	118 (77.1)	112 (83)	0.218	0.961
**First 24 months[2]**	141 (92.2)	132 (97)	0.071	0.254
**Infant hospitalization**				
**Month 1**	10 (5.6)	10 (6.3)	0.796	0.855
**Months 2 and 3**	7 (4.1)	7 (4.5)	0.870	0.879
**Months 4 to 6**	9 (5.6)	4 (2.7)	0.207	0.243

	Control	Experimental	Unadjusted Analysis	Adjusted Analysis[1]
**First 6 months**	19 (11.8)	17 (11.5)	0.931	0.771
**Months 7 to 12**	20 (12.7)	8 (5.6)	**0.035**	0.085
**First 12 months**	33 (20.9)	22 (15.4)	0.217	0.256
**Months 13 to 24**	15 (9.8)	18 (13.2)	0.360	0.307
**First 24 months**	43 (28.1)	34 (25)	0.551	0.647
**CONTINUOUS VARIABLES**	***Mean (SD)***	***Mean (SD)***	***p-Value***	***p-Value***
**Number of doctor visits for infant illnesses**				
**First month**	0.25 (0.495)	0.26 (0.666)	0.939	0.998I
**Months 2 and 3**	0.45 (1.091)	0.34 (0.739)	0.278	0.664
**Months 4 to 6**	0.83 (1.551)	0.54 (0.999)	0.054	0.084
**First 6 months**	1.50 (1.982)	1.13 (1.527)	0.062	0.134
**Months 7 to 12**	1.49 (2.312)	1.13 (1.448)	0.096	0.154
**First 12 months**	2.97 (3.095)	2.22 (2.302)	**0.016**	**0.045**
**Months 13 to 24[2]**	2.77 (3.876)	2.83 (3.990)	0.900	0.994
**First 24 months[2]**	5.75 (5.718)	5.04 (5.069)	0.275	0.322
**Number of infant hospitalizations**				
**First month**	0.06 (0.230)	0.06 (0.243)	0.797	0.858
**Months 2 and 3**	0.04 (0.199)	0.06 (0.283)	0.542	0.446
**Months 4 to 6**	0.06 (0.230)	0.03 (0.163)	0.202	0.238
**First 6 months**	0.12 (0.349)	0.14 (0.398)	0.789	0.887
**Months 7 to 12**	0.15 (0.420)	0.08 (0.337)	0.117	0.192
**First 12 months**	0.27 (0.583)	0.21 (0.567)	0.349	0.401
**Months 13 to 24**	0.17 (0.667)	0.16 (0.443)	0.904	0.999
**First 24 months**	0.45 (1.026)	0.37 (0.758)	0.438	0.527
**Total cost of formula and water (USD)**				
**First month**	22.91 (33.929)	22.50 (36.593)	0.915	0.373

	Control	Experimental	Unadjusted Analysis	Adjusted Analysis[1]
**Months 2 and 3**	87.94 (105.561)	94.54 (110.771)	0.581	0.782
**Months 4 to 6**	173.51 (167.921)	171.09 (174.993)	0.902	0.411
**First 6 months**	284.51 (282.091)	283.57 (286.489)	0.977	0.422
**Months 7 to 12**	303.92 (278.559)	367.46 (306.804)	0.061	0.473
**First 12 months**	581.08 (486.212)	641.56 (528.693)	0.302	0.938
**Months 13 to 24[2,3]**	559.98 (396.350)	583.43 (395.096)	0.617	0.942
**First 24 months[2,3]**	1156.11 (722.304)	1224.45 (729.816)	0.427	0.826
**Total cost of doctor visits for infant illnesses (USD)**				
**First month**	9.02 (21.402)	10.38 (33.172)	0.649	0.955
**Months 2 and 3**	19.69 (54.461)	13.98 (35.543)	0.266	0.266
**Months 4 to 6**	33.45 (63.808)	26.44 (56.773)	0.311	0.299
**First 6 months**	61.52 (85.898)	51.35 (82.856)	0.291	0.194
**Months 7 to 12**	64.01 (103.932)	55.37 (78.547)	0.420	0.328
**First 12 months**	125.02 (141.429)	102.92 (120.910)	0.148	0.101
**Months 13 to 24[2]**	133.97 (221.802)	144.40 (217.761)	0.688	0.840
**First 24 months[2]**	259.59 (308.066)	249.44 (265.564)	0.766	0.398
**Total cost of infant hospitalizations (USD)**				
**First month**	793.82 (4,293.041)	886.00 (4,492.518)	0.847	0.735
**Months 2 and 3**	604.22 (3,049.150)	948.43 (5,050.373)	0.451	0.447
**Months 4 to 6**	628.34 (2,603.148)	515.62 (3,775.232)	0.759	0.876
**First 6 months**	1,526.38 (4,535.873)	1,771.35 (5,643.984)	0.673	0.730
**Months 7 to 12**	1,835.65 (6,567.496)	982.74 (4,709.101)	0.193	0.319
**First 12 months**	3,391.02 (8,298.904)	2,714.96 (8,077.900)	0.475	0.577
**Months 13 to 24**	2,876.90 (1,3049.830)	2,780.44 (9,805.611)	0.944	0.998
**First 24 months**	6,378.74 (18,766.041)	5,481.24 (12,630.570)	0.638	0.726
**Total cost of infant medications (USD)**				
**First month**	2.61 (12.960)	4.19 (21.314)	0.404	0.448

	Control	Experimental	Unadjusted Analysis	Adjusted Analysis[1]
**Months 2 and 3**	6.07 (19.648)	4.01 (13.848)	0.275	0.318
**Months 4 to 6**	12.92 (40.289)	6.77 (15.313)	0.073	0.088
**First 6 months**	20.91 (55.627)	13.64 (22.971)	0.140	0.159
**Months 7 to 12**	28.05 (64.557)	18.67 (52.137)	0.169	0.251
**First 12 months**	43.75 (71.534)	31.16 (59.276)	0.099	0.161
**Months 13 to 24**[2]	28.39 (39.335)	31.20 (59.433)	0.633	0.583
**First 24 months**[2]	72.15 (92.296)	62.04 (94.675)	0.360	0.444
**Total cost of maternal non-routine doctor visits due to BF (USD)**				
**First month**	3.49 (17.443)	3.88 (21.634)	0.853	0.513
**Months 2 and 3**	2.79 (12.502)	2.18 (13.200)	0.665	0.584
**Months 4 to 6**	2.75 (17.748)	1.25 (9.268)	0.358	0.208
**First 6 months**	8.98 (36.528)	7.76 (28.226)	0.745	0.300
**Months 7 to 12**	1.47 (16.060)	0.09 (1.111)	0.283	0.332
**First 12 months**	10.62 (45.280)	8.12 (28.676)	0.573	0.241
**Months 13 to 24**	8.87 (49.056)	2.18 (15.552)	0.112	0.091
**First 24 months**	19.84 (84.309)	10.72 (32.644)	0.218	0.094
**Overall total cost**[4] **(USD)**				
**First month**	831.84 (4308.943)	970.56 (4496.961)	0.772	0.793
**First 6 months**	1902.30 (4601.112)	2389.28 (5706.914)	0.408	0.510
**First 12 months**	4151.48 (8372.374)	3760.34 (8180.059)	0.683	0.725
**First 24 months**[3,5]	7862.77 (18922.603)	7373.06 (12843.583)	0.801	0.838

BF: Breastfeeding.

[1] Variables: Adjusted for the number of children and household income.

[2] Missing data for 1 participant in the intervention group.

[3] Missing data for 1 participant in the control group.

[4] The sum of the costs of formula and water, doctor visits for infant illnesses, infant hospitalizations, infant medications, maternal non-routine doctor visits due to BF, and implementation cost.

[5] Missing data for 2 participants in the intervention group.

*Chi Square test.

£Independent Student's t test.
