# Supplementary material for: Cost-benefit analysis of a multicomponent breastfeeding promotion and support intervention in a developing country
Source: PLoS One. 2024 Jul 19;19(7):e0295194. doi: 10.1371/journal.pone.0295194 (PMC11259277; doi:10.1371/journal.pone.0295194)
Supplement: S2 File — (PDF) [file pone.0295194.s002.pdf]

S2 File. Comparison of the Exclusive/Predominant BF and any formula groups at different time points.

	EBF/Predominant BF[1]	Any FF[2]	*p*-Value
**CATEGORICAL VARIABLES**	***n (%)***	***n (%)***	
**Doctor visit for infant illness**			
**Month 1**	26 (14.9)	44 (26.8)	**0.007**
**Months 2 and 3**	31 (22.1)	58 (30.9)	0.079
**Months 4 to 6**	44 (41.9)	93 (45.6)	0.537
**First 6 months**	54 (62.1)	148 (66.7)	0.445
**Months 7 to 12**	38 (55.9)	154 (66.1)	0.123
**First 12 months**	45 (80.4)	210 (85.7)	0.315
**Months 13 to 24[3]**	13 (68.4)	216 (80.9)	0.231
**First 24 months[3]**	13 (86.7)	257 (94.8)	0.201
**Infant hospitalization**			
**Month 1**	6 (3.4)	14 (8.5)	**0.046**
**Months 2 and 3**	4 (2.9)	10 (5.3)	0.275
**Months 4 to 6**	5 (4.8)	8 (3.9)	0.768
**First 6 months**	6 (6.9)	30 (13.5)	0.103
**Months 7 to 12**	6 (8.8)	22 (9.4)	0.877
**First 12 months**	9 (16.1)	46 (18.8)	0.637
**Months 13 to 24**	0 (0)	33 (12.3)	0.143
**First 24 months**	3 (20)	73 (26.8)	0.766
**CONTINUOUS VARIABLES**	*Mean (SD)*	*Mean (SD)*	
**Number of doctor visits for infant illnesses**			
**First month**	0.15 (0.378)	0.36 (0.725)	**0.001**
**Months 2 and 3**	0.28 (0.635)	0.48 (1.107)	**0.035**
**Months 4 to 6**	0.66 (1.200)	0.71 (1.383)	0.759
**First 6 months**	1.14 (1.579)	1.40 (1.858)	0.253

	EBF/Predominant BF[1]	Any FF[2]	*p*-Value
**Months 7 to 12**	1.04 (1.616)	1.40 (2.040)	0.188
**First 12 months**	2.45 (2.763)	2.65 (2.774)	0.615
**Months 13 to 24**[3]	2.05 (3.325)	2.87 (3.969)	0.380
**First 24 months**[3]	4.73 (5.035)	5.49 (5.459)	0.602
**Number of infant hospitalizations**			
**First month**	0.03 (0.182)	0.09 (0.280)	**0.049**
**Months 2 and 3**	0.03 (0.167)	0.06 (0.285)	0.162
**Months 4 to 6**	0.05 (0.214)	0.04 (0.195)	0.728
**First 6 months**	0.07 (0.255)	0.15 (0.408)	**0.030**
**Months 7 to 12**	0.13 (0.486)	0.11 (0.349)	0.636
**First 12 months**	0.25 (0.640)	0.24 (0.561)	0.914
**Months 13 to 24**	0.00 (0.000)	0.18 (0.591)	**<0.001**
**First 24 months**	0.27 (0.594)	0.42 (0.926)	0.529
**Total cost of formula and water (USD)**			
**First month**	1.21 (3.711)	45.67 (39.014)	**<0.001**
**Months 2 and 3**	1.72 (7.043)	157.65 (99.575)	**<0.001**
**Months 4 to 6**	2.32 (18.651)	259.87 (146.880)	**<0.001**
**First 6 months**	3.95 (21.448)	393.83 (262.745)	**<0.001**
**Months 7 to 12**	2.34 (19.263)	430.93 (263.903)	**<0.001**
**First 12 months**	7.00 (32.539)	747.60 (461.743)	**<0.001**
**Months 13 to 24**	7.20 (31.384)	610.98 (378.209)	**<0.001**
**First 24 months**	0.00 (0.000)	1,253.79 (687.633)	**<0.001**
**Total cost of doctor visits for infant illnesses (USD)**			
**First month**	4.92 (13.604)	14.72 (36.429)	**0.001**
**Months 2 and 3**	13.98 (47.705)	19.17 (45.402)	0.317
**Months 4 to 6**	29.81 (61.086)	30.24 (60.414)	0.954
**First 6 months**	48.96 (90.624)	59.67 (81.952)	0.317
**Months 7 to 12**	50.68 (95.351)	62.60 (91.936)	0.352

	EBF/Predominant BF[1]	Any FF[2]	*p*-Value
**First 12 months**	108.61 (151.377)	115.87 (127.880)	0.712
**Months 13 to 24[3]**	116.84 (215.896)	141.47 (220.656)	0.638
**First 24 months[3]**	257.85 (316.368)	256.21 (287.988)	0.983
**Total cost of infant hospitalizations (USD)**			
**First month**	530.99 (3,921.923)	1,164.21 (4,815.633)	0.187
**Months 2 and 3**	370.19 (2,171.280)	1,065.95 (5,105.865)	0.095
**Months 4 to 6**	530.61 (2,395.956)	596.87 (3,567.072)	0.864
**First 6 months**	783.59 (2,910.246)	1,980.78 (5,692.396)	**0.016**
**Months 7 to 12**	1,962.58 (8,837.540)	1,275.15 (4,509.355)	0.388
**First 12 months**	3,417.18 (10,529.932)	2,990.44 (7,576.485)	0.726
**Months 13 to 24**	0.00 (0.000)	3,053.38 (12,036.876)	**<0.001**
**First 24 months**	2,963.46 (6,737.978)	6,127.49 (16,557.148)	0.462
**Total cost of infant medications (USD)**			
**First month**	1.11 (4.550)	5.75 (24.384)	**0.017**
**Months 2 and 3**	2.96 (9.275)	6.67 (21.046)	**0.032**
**Months 4 to 6**	8.57 (20.556)	10.69 (35.292)	0.571
**First 6 months**	12.45 (23.143)	19.38 (48.865)	0.206
**Months 7 to 12**	28.66 (100.328)	22.12 (39.950)	0.600
**First 12 months**	44.78 (112.841)	36.17 (50.021)	0.578
**Months 13 to 24[3]**	6.54 (8.748)	31.58 (51.108)	**<0.001**
**First 24 months[3]**	33.24 (41.572)	69.72 (95.328)	0.142
**Total cost of maternal non-routine doctor visits due to BF (USD)**			
**First month**	1.88 (12.646)	5.59 (24.719)	0.086
**Months 2 and 3**	1.45 (8.959)	3.28 (15.050)	0.172
**Months 4 to 6**	5.41 (23.842)	0.29 (3.338)	**0.031**
**First 6 months**	8.04 (38.595)	8.53 (30.278)	0.907
**Months 7 to 12**	0.00 (0.000)	1.06 (13.254)	0.513
**First 12 months**	4.02 (24.636)	10.67 (40.677)	0.115

	EBF/Predominant BF[1]	Any FF[2]	*p*-Value
**Months 13 to 24**	3.57 (15.548)	5.92 (38.567)	0.792
**First 24 months**	7.57 (20.415)	16.10 (67.176)	0.625
**Total cost[4] (USD)**			
**First month**	540.11 (3,921.060)	1,235.94 (4,831.453)	0.148
**First 6 months**	857.00 (2,940.275)	2,462.19 (5,737.168)	**0.001**
**First 12 months**	3,581.60 (10,659.165)	3,900.74 (7,651.538)	0.795
**First 24 months**	3,262.13 (6,846.196)	7,745.92 (16,678.377)	0.302

[1] EBF/Predominant BF refers to the group of infants who are exclusively breastfed or receiving, in addition to breastmilk, a small quantity of formula milk (a maximum of two formula bottles per week).

[2] Any FF refers to the group of infants receiving formula milk, with or without breastmilk.

[3] Missing data for 1 participant in the any FF group.

[4] The sum of the costs of formula and water, doctor visits for infant illnesses, infant hospitalizations, infant medications, and maternal non-routine doctor visits due to BF.
