# Supplementary material for: Cost-benefit analysis of a multicomponent breastfeeding promotion and support intervention in a developing country
Source: PLoS One. 2024 Jul 19;19(7):e0295194. doi: 10.1371/journal.pone.0295194 (PMC11259277; doi:10.1371/journal.pone.0295194)
Supplement: S3 File — (PDF) [file pone.0295194.s003.pdf]

S3 File. Comparison of the exclusive formula feeding and any breastfeeding groups at different time points.

	Any BF[1]	EFF[2]	*p*-Value
**CATEGORICAL VARIABLES**	***n (%)***	***n (%)***	
**Doctor visit for infant illness**			
**Month 1**	63 (19.6)	7 (38.9)	0.068
**Months 2 and 3**	62 (24.4)	27 (36.5)	**0.04**
**Months 4 to 6**	78 (41.8)	59 (48)	0.296
**First 6 months**	192 (65.8)	10 (58.8)	0.559
**Months 7 to 12**	72 (61.5)	120 (65.2)	0.517
**First 12 months**	243 (85)	12 (80)	0.710
**Months 13 to 24[3]**	17 (73.9)	212 (80.6)	0.422
**First 24 months[4]**	257 (94.8)	13 (86.7)	0.201
**Infant hospitalization**			
**Month 1**	17 (5.3)	3 (16.7)	0.081
**Months 2 and 3**	6 (2.4)	8 (10.8)	**0.004**
**Months 4 to 6**	7 (3.8)	6 (4.9)	0.633
**First 6 months**	31 (10.6)	5 (29.4)	**0.035**
**Months 7 to 12**	10 (8.5)	18 (9.8)	0.719
**First 12 months**	50 (17.5)	5 (33.3)	0.162
**Months 13 to 24**	0 (0)	33 (12.5)	0.088
**First 24 months**	70 (25.7)	6 (40)	0.236
**CONTINUOUS VARIABLES**	***Mean (SD)***	***Mean (SD)***	
**Number of doctor visits for infant illnesses**			
**First month**	0.24 (0.573)	0.44 (0.405)	0.249
**Months 2 and 3**	0.35 (0.950)	0.54 (0.894)	0.134
**Months 4 to 6**	0.67 (1.306)	0.72 (1.351)	0.712
**First 6 months**	1.30 (1.732)	1.76 (2.562)	0.469

	Any BF[1]	EFF[2]	*p*-Value
**Months 7 to 12**	1.17 (1.753)	1.41 (2.073)	0.296
**First 12 months**	2.59 (2.729)	3.00 (3.525)	0.667
**Months 13 to 24[3]**	2.00 (3.030)	2.89 (3.995)	0.299
**First 24 months[4]**	5.41 (5.453)	6.07 (5.161)	0.651
**Number of infant hospitalizations**			
**First month**	0.05 (0.224)	0.17 (0.383)	0.229
**Months 2 and 3**	0.02 (0.152)	0.14 0.416)	**0.027**
**Months 4 to 6**	0.04 (0.191)	0.05 (0.216)	0.634
**First 6 months**	0.11 (0.324)	0.47 (0.800)	0.082
**Months 7 to 12**	0.12 (0.439)	0.11 (0.345)	0.810
**First 12 months**	0.22 (0.511)	0.73 (1.223)	0.125
**Months 13 to 24**	0.00 (0.000)	0.18 (0.596)	**<0.001**
**First 24 months**	0.39 (5.453)	0.80 (1.207)	0.214
**Total cost of formula and water (USD)**			
**First month**	18.54 (29.399)	97.24 (45.827)	**<0.001**
**Months 2 and 3**	47.99 (72.544)	239.08 (72.910)	**<0.001**
**Months 4 to 6**	64.22 (96.387)	335.87 (122.516)	**<0.001**
**First 6 months**	260.37 (269.821)	690.96 (198.893)	**<0.001**
**Months 7 to 12**	90.82 (150.952)	488.80 (254.793)	**<0.001**
**First 12 months**	580.36 (499.955)	1171.45 (248.449)	**<0.001**
**Months 13 to 24**	92.45 (219.292)	612.70 (379.582)	**<0.001**
**First 24 months**	1,152.70 (722.094)	1,833.05 (431.095)	**<0.001**
**Total cost of doctor visits for infant illnesses (USD)**			
**First month**	8.55 (21.939)	29.53 (74.801)	0.251
**Months 2 and 3**	14.41 (42.873)	25.69 (56.331)	0.114
**Months 4 to 6**	29.93 (65.000)	30.34 (53.361)	0.953
**First 6 months**	54.89 (82.022)	86.81 (118.224)	0.288
**Months 7 to 12**	50.42 (83.347)	65.93 (97.917)	0.157

	Any BF[1]	EFF[2]	*p*-Value
**First 12 months**	114.15 (133.050)	121.64 (121.593)	0.831
**Months 13 to 24[3]**	112.70 (195.985)	142.21 (222.216)	0.538
**First 24 months[4]**	256.32 (292.723)	255.86 (216.948)	0.995
**Total cost of infant hospitalizations (USD)**			
**First month**	755.43 (4,319.981)	2,297.90 (5,301.932)	0.241
**Months 2 and 3**	357.73 (2,398.332)	2,180.57 (7,331.256)	**0.038**
**Months 4 to 6**	428.14 (2,186.348)	795.45 (4,328.696)	0.385
**First 6 months**	1,412.07 (4,625.424)	5,622.44 (9,580.301)	0.090
**Months 7 to 12**	1,573.39 (7,173.252)	1,339.56 (4,705.231)	0.732
**First 12 months**	2,763.17 (7,592.472)	8,916.94 (14,986.423)	0.136
**Months 13 to 24**	0.00 (0.000)	3,099.64 (12,122.130)	**<0.001**
**First 24 months**	5,758.87 (16,276.402)	9,647.84 (14,786.489)	0.366
**Total cost of infant medications (USD)**			
**First month**	3.44 (17.860)	1.92 (3.674)	0.720
**Months 2 and 3**	4.68 (16.777)	6.46 (18.321)	0.431
**Months 4 to 6**	7.99 (18.920)	12.97 (43.328)	0.231
**First 6 months**	17.73 (44.178)	12.24 (22.809)	0.612
**Months 7 to 12**	25.81 (79.301)	22.19 (41.587)	0.604
**First 12 months**	38.29 (67.366)	27.79 (37.095)	0.550
**Months 13 to 24[3]**	11.19 (17.581)	31.55 (51.383)	**<0.001**
**First 24 months[4]**	68.79 (95.095)	50.07 (59.828)	0.452
**Total cost of maternal non-routine doctor visits due to BF (USD)**			
**First month**	3.88 (20.026)	0.00 (0.000)	0.412
**Months 2 and 3**	1.68 (9.707)	5.31 (19.996)	0.135
**Months 4 to 6**	3.13 (18.092)	0.37 (4.134)	**0.047**
**First 6 months**	8.88 (33.647)	0.00 (0.000)	**<0.001**
**Months 7 to 12**	1.82 (18.459)	0.18 (2.449)	0.342
**First 12 months**	9.93 (39.190)	0.00 (0.000)	**<0.001**

	Any BF[1]	EFF[2]	*p*-Value
**Months 13 to 24**	2.95 (14.132)	6.01 (38.853)	0.708
**First 24 months**	16.52 (67.258)	0.00 (0.000)	0.343
**Total cost[5] (USD)**			
**First month**	789.83 (4,328.556)	2,426.58 (5,312.480)	0.215
**First 6 months**	1,753.95 (4,661.405)	6,412.45 (9,705.333)	0.066
**First 12 months**	3,505.89 (7,654.094)	10,237.82 (15,109.551)	0.108
**First 24 months**	7,274.07 (16,406.194)	11,786.82 (14,841.658)	0.298

[1] Any BF refers to the group of infants receiving breastmilk, with or without formula milk.

[2] EFF refers to the group of infants receiving formula milk exclusively.

[3] Missing data for 1 participant in the EFF group.

[4] Missing data for 1 participant in the any BF group.

[5] The sum of the costs of formula and water, doctor visits for infant illnesses, infant hospitalizations, infant medications, and maternal non-routine doctor visits due to BF.
