# Supplementary material for: Cost-benefit analysis of a multicomponent breastfeeding promotion and support intervention in a developing country
Source: PLoS One. 2024 Jul 19;19(7):e0295194. doi: 10.1371/journal.pone.0295194 (PMC11259277; doi:10.1371/journal.pone.0295194)
Supplement: S4 File — (PDF) [file pone.0295194.s004.pdf]

S4 File. Comparison of the Exclusive/Predominant BF, mixed feeding, and exclusive formula feeding groups.

	EBF/Predominant BF[1]	Mixed Feeding[2]	EFF[3]	*p*-Value
**CATEGORICAL VARIABLES**	***n (%)***	***n (%)***	***n (%)***	
**Doctor visit for infant illness**				
**Month 1**	26 (14.9)	37 (25.3)	7 (38.9)	**0.009**
**Months 2 and 3**	31 (22.1)	31 (27.2)	27 (36.5)	0.081
**Months 4 to 6**	44 (41.9)	34 (42)	59 (48)	0.579
**First 6 months**	54 (62.1)	138 (67.3)	10 (58.8)	0.582
**Months 7 to 12**	38 (55.9)	34 (69.4)	120 (65.2)	0.263
**First 12 months**	45 (80.4)	198 (86.1)	12 (80)	0.458
**Months 13 to 24[4]**	13 (68.4)	4 (100)	212 (80.6)	0.369
**First 24 months[5]**	13 (86.7)	244 (95.3)	13 (86.7)	0.108
**Infant hospitalization**				
**Month 1**	6 (3.4)	11 (7.5)	3 (16.7)	**0.039**
**Months 2 and 3**	4 (2.9)	2 (1.8)	8 (10.8)	**0.011**
**Months 4 to 6**	5 (4.8)	2 (2.5)	6 (4.9)	0.928
**First 6 months**	6 (6.9)	25 (12.2)	5 (29.4)	**0.033**
**Months 7 to 12**	6 (8.8)	4 (8.2)	18 (9.8)	1.000
**First 12 months**	9 (16.1)	41 (17.8)	5 (33.3)	0.282
**Months 13 to 24**	0 (0)	0 (0)	33 (12.5)	0.233
**First 24 months**	3 (20)	67 (26.1)	6 (40)	0.383
**CONTINUOUS VARIABLES**	*Mean (SD)*	*Mean (SD)*	*Mean (SD)*	
**Number of doctor visits for infant illnesses**				
**First month**	0.15 (0.378)	0.35 (0.730)	0.44 (0.705)	**0.004**
**Months 2 and 3**	0.28 (0.635)	0.45 (1.227)	0.54 (0.894)	0.117

	EBF/Predominant BF[1]	Mixed Feeding[2]	EFF[3]	*p*-Value
**Months 4 to 6**	0.66 (1.200)	0.68 (1.439)	0.72 (1.351)	0.928
**First 6 months**	1.14 (1.579)	1.37 (1.793)	1.76 (2.562)	0.352
**Months 7 to 12**	1.04 (1.616)	1.35 (1.932)	1.41 (2.073)	0.412
**First 12 months**	2.45 (2.763)	2.63 (2.726)	3.00 (3.525)	0.778
**Months 13 to 24[4]**	2.05 (3.325)	1.75 (0.957)	2.89 (3.995)	0.577
**First 24 months[5]**	4.73 (5.035)	5.45 (5.483)	6.07 (5.161)	0.798
**Number of infant hospitalizations**				
**First month**	0.03 (0.182)	0.08 (0.265)	0.17 (0.383)	**0.041**
**Months 2 and 3**	0.03 (0.167)	0.02 (0.132)	0.14 0.416)	**0.002**
**Months 4 to 6**	0.05 (0.214)	0.02 (0.156)	0.05 (0.216)	0.664
**First 6 months**	0.07 (0.255)	0.13 (0.348)	0.47 (0.800)	**<0.001**
**Months 7 to 12**	0.13 (0.486)	0.10 (0.368)	0.11 (0.345)	0.889
**First 12 months**	0.25 (0.640)	0.21 (0.476)	0.73 (1.223)	**0.003**
**Months 13 to 24**	0.00 (0.000)	0.00 (0.000)	0.18 (0.596)	0.346
**First 24 months**	0.27 (0.594)	0.40 (0.905)	0.80 (1.207)	0.205
**Total cost of formula and water (USD)**				
**First month**	1.21 (3.711)	39.31 (33.076)	97.24 (45.827)	**<0.001**
**Months 2 and 3**	1.72 (7.043)	104.80 (76.250)	239.08 (72.910)	**<0.001**
**Months 4 to 6**	2.32 (18.651)	144.46 (97.393)	335.87 (122.516)	**<0.001**
**First 6 months**	3.95 (21.448)	369.19 (252.424)	690.96 (198.893)	**<0.001**
**Months 7 to 12**	2.34 (19.263)	213.62 (167.523)	488.80 (254.793)	**<0.001**
**First 12 months**	7.00 (32.539)	719.96 (459.199)	1171.45 (248.449)	**<0.001**
**Months 13 to 24[6]**	7.20 (31.384)	497.39 (286.495)	612.70 (379.582)	**<0.001**
**First 24 months**	0.00 (0.000)	1,219.98 (685.254)	1833.05 (431.095)	**<0.001**
**Total cost of doctor visits for infant illnesses (USD)**				
**First month**	4.92 (13.604)	12.90 (28.377)	29.53 (74.801)	**<0.001**
**Months 2 and 3**	13.98 (47.705)	14.94 (36.269)	25.69 (56.331)	0.182

	EBF/Predominant BF[1]	Mixed Feeding[2]	EFF[3]	*p*-Value
**Months 4 to 6**	29.81 (61.086)	30.07 (70.137)	30.34 (53.361)	0.998
**First 6 months**	48.96 (90.624)	57.42 (78.186)	86.81 (118.224)	0.235
**Months 7 to 12**	50.68 (95.351)	50.06 (64.007)	65.93 (97.917)	0.368
**First 12 months**	108.61 (151.377)	115.50 (128.523)	121.64 (121.593)	0.920
**Months 13 to 24[4]**	116.84 (215.896)	93.02 (37.193)	142.21 (222.216)	0.812
**First 24 months[5]**	257.85 (316.368)	256.23 (291.945)	255.86 (216.948)	1.000
**Total cost of infant hospitalizations (USD)**				
**First month**	530.99 (3,921.923)	1,024.44 (4,753.335)	2,297.90 (5,301.932)	0.210
**Months 2 and 3**	370.19 (2,171.280)	342.43 (2,660.588)	2,180.57 (7,331.256)	**0.004**
**Months 4 to 6**	530.61 (2,395.956)	295.31 (1,886.688)	795.45 (4,328.696)	0.547
**First 6 months**	783.59 (2,910.246)	1,678.79 (5,168.004)	5,622.44 (9,580.301)	**0.001**
**Months 7 to 12**	1,962.58 (8,837.540)	1,033.29 (3,714.976)	1,339.56 (4,705.231)	0.653
**First 12 months**	3,417.18 (10,529.932)	2,603.93 (6,706.853)	8,916.94 (14,986.423)	**0.014**
**Months 13 to 24**	0.00 (0.000)	0.00 (0.000)	3,099.64 (12,122.130)	0.475
**First 24 months**	2,963.46 (6,737.978)	5,922.02 (16,657.628)	9647.84 (14786.489)	0.526
**Total cost of infant medications (USD)**				
**First month**	1.11 (4.550)	6.22 (25.783)	1.92 (3.674)	**0.030**
**Months 2 and 3**	2.96 (9.275)	6.860 (22.719)	6.46 (18.321)	0.151
**Months 4 to 6**	8.57 (20.556)	7.23 (16.658)	12.97 (43.328)	0.370
**First 6 months**	12.45 (23.143)	19.97 (50.412)	12.24 (22.809)	0.351
**Months 7 to 12**	28.66 (100.328)	21.86 (33.469)	22.19 (41.587)	0.725
**First 12 months**	44.78 (112.841)	36.71 (50.764)	27.79 (37.095)	0.600
**Months 13 to 24[4]**	6.54 (8.748)	33.31 (31.913)	31.55 (51.383)	0.106
**First 24 months[5]**	33.24 (41.572)	70.87 (96.961)	50.07 (59.828)	0.240
**Total cost of maternal non-routine doctor visits due to BF (USD)**				
**First month**	1.88 (12.646)	6.28 (26.125)	0.00 (0.000)	0.094
**Months 2 and 3**	1.45 (8.959)	1.96 (10.588)	5.31 (19.996)	0.096

	EBF/Predominant BF[1]	Mixed Feeding[2]	EFF[3]	*p*-Value
**Months 4 to 6**	5.41 (23.842)	0.16 (1.477)	0.37 (4.134)	**0.011**
**First 6 months**	8.04 (38.595)	9.24 (31.410)	0.00 (0.000)	0.534
**Months 7 to 12**	0.00 (0.000)	4.34 (28.500)	0.18 (2.449)	0.068
**First 12 months**	4.02 (24.636)	11.37 (41.893)	0.00 (0.000)	0.271
**Months 13 to 24**	3.57 (15.548)	0.00 (0.000)	6.01 (38.853)	0.918
**First 24 months**	7.57 (20.415)	17.04 (68.999)	0.00 (0.000)	0.551
**Total cost[7] (USD)**				
**First month**	540.11 (3,921.060)	1,089.15 (4,768.036)	2,426.58 (5,312.480)	0.165
**First 6 months**	857.00 (2,940.275)	2,134.60 (5,182.864)	6,412.45 (9,705.333)	**<0.001**
**First 12 months**	3,581.60 (10,659.165)	3,487.46 (6,754.390)	10,237.82 (15,109.551)	**0.009**
**First 24 months**	3,262.13 (6,846.196)	7,509.15 (16,775.601)	11,786.82 (14,841.658)	0.361

[1] EBF/Predominant BF refers to the group of infants who are exclusively breastfed or receiving, in addition to breastmilk, a small quantity of formula milk (a maximum of two formula bottles per week).

[2] Mixed feeding refers to the group of infants receiving both breast milk and formula milk (more than two formula bottles per week).

[3] EFF refers to the group of infants receiving formula milk exclusively.

[4] Missing data for 1 participant in the EFF group.

[5] Missing data for 1 participant in the mixed feeding group.

[6] Missing data for 1 participant in the EBF group.

[7] The sum of the costs of formula and water, doctor visits for infant illnesses, infant hospitalizations, infant medications, and maternal non-routine doctor visits due to BF.
